# Intrapartum Febrile Morbidity and Maternal Birth Trauma After Vaginal Delivery: A Parity-Stratified Retrospective Cohort Study

**DOI:** 10.3390/jcm15166220

**Published:** 2026-08-11

**Authors:** Eiman Shalabna, Amy Goh, Deepa Gopinath, Chen Nahshon, Esther Maor-Sagie, Rinat Gabbay-Benziv

**Affiliations:** 1 The Ruth and Bruce Rappaport Faculty of Medicine, Technion—Israel Institute of Technology, Haifa 3200003, Israel; eiman.shalabna@health.nsw.gov.au (E.S.);; 2 Department of Obstetrics and Gynecology, Westmead Hospital, The University of Sydney, Sydney, NSW 2145, Australia; 3 Department of Obstetrics and Gynecology, Nepean Hospital, Sydney, NSW 2747, Australia; 4 Department of Obstetrics and Gynecology, Hillel Yaffe Medical Center, Hadera 3820302, Israel

**Keywords:** intrapartum fever, maternal birth trauma, perineal lacerations, chorioamnionitis, vaginal delivery

## Abstract

**Objective:** We aimed to evaluate the association between intrapartum febrile morbidity and maternal birth trauma among women undergoing vaginal delivery, with stratification by parity. **Methods:** This retrospective cohort study included women with singleton, cephalic, vaginal deliveries at a university-affiliated medical center between 2018 and 2024, excluding multiple gestations and cesarean deliveries. The primary exposure was intrapartum febrile morbidity, defined as maternal intrapartum temperature ≥38.0 °C, with or without clinical chorioamnionitis. The primary outcome was maternal birth trauma, including first- through fourth-degree perineal lacerations, cervical lacerations, or episiotomy. Multivariable logistic regression models were used to assess the independent association between intrapartum febrile morbidity and birth trauma. Analyses were additionally stratified by parity. **Results:** Among 21,736 women with vaginal deliveries included in the study, birth trauma occurred in 41.8% of deliveries (9075/21,736). Intrapartum febrile morbidity was more common among women with birth trauma compared with those delivering with an intact perineum (4.2% vs. 1.8%, *p* < 0.001). After adjustment for confounders, intrapartum febrile morbidity remained independently associated with birth trauma in the overall cohort (adjusted odds ratio [aOR], 1.5; 95% CI, 1.1–1.8). In parity-stratified analyses, the association persisted among multiparous women (aOR 1.8; 95% CI, 1.2–2.9) but was not statistically significant among nulliparous women (aOR, 1.3; 95% CI, 0.9–1.7). Nulliparity and operative vaginal delivery were the strongest predictors of birth trauma. **Conclusions**: These findings suggest that intrapartum febrile morbidity may identify women at increased risk of lower genital tract trauma beyond established mechanical risk factors.

## 1. Introduction

Maternal birth trauma remains a common complication of vaginal delivery, occurring in 50–80% of births, and encompassing a spectrum of injuries from first- and second-degree lacerations to obstetric anal sphincter injury (OASIS), cervical tears and episiotomies, all of which may be associated with significant short and long-term morbidity, including pain, dyspareunia, and anal incontinence [1,2,3,4,5]. Second-degree lacerations, involving the perineal musculature, and episiotomy, an iatrogenic perineal incision, require surgical repair and are associated with clinically meaningful postpartum pain and early functional limitations, such as impaired mobility and difficulty with personal hygiene; however, even minor tears may cause discomfort and interfere with early postpartum daily functioning [6,7,8]. In contemporary obstetric practice, episiotomy is generally not performed routinely, but rather selectively, most commonly when there is concern for impending severe perineal tearing or compromised perineal tissue integrity during delivery [9].

Established risk factors for birth trauma include nulliparity, operative vaginal delivery, prolonged second stage of labor and macrosomia [10,11]. These factors largely reflect the mechanical forces exerted on the perineal tissues during childbirth. However, childbirth-related tissue injury is likely influenced not only by mechanical stress, but also by the biological properties of the tissue at the delivery time.

Clinical chorioamnionitis represents the most frequent infection-related diagnosis in labor and delivery units, affecting approximately 1% to 6% of term gestations [12]. Maternal intrapartum fever, the cardinal clinical sign of this condition, reflects activation of inflammatory pathways within the intrauterine and maternal tissues.

Inflammation plays a central role in extracellular matrix remodeling, collagen degradation, and tissue repair processes [13]. In other surgical and wound-healing settings, infected or inflamed tissue is known to be more susceptible to mechanical injury and to demonstrate impaired healing [13]. It is therefore biologically plausible that intrapartum inflammatory activation, though classically localized to the uterus and adjacent reproductive tissues, may extend to or otherwise influence perineal tissue integrity, potentially rendering it more vulnerable to laceration during vaginal delivery and affecting subsequent healing; this extension to the perineum remains to be directly demonstrated.

Despite this biologic plausibility, the potential contribution of intrapartum fever and inflammatory processes to birth trauma has received limited attention, and fever is not routinely incorporated into predictive models for perineal injury cervical tears, or OASIS [14,15,16]. Moreover, most existing studies of birth trauma have focused primarily on mechanical and obstetric risk factors, while the potential contribution of inflammatory processes during labor has remained largely unexplored.

Given the clinical burden of birth trauma and the frequency of intrapartum fever during labor, understanding whether an inflammatory intrapartum environment contributes to perineal injury may provide important insights into mechanisms of obstetric trauma. Because episiotomy is often performed selectively in response to suspected impending severe perineal tearing, its occurrence may also reflect increased vulnerability of the perineal tissues during labor. This study therefore aimed to evaluate the association between intrapartum febrile morbidity and maternal birth trauma in a large, contemporary cohort of vaginal deliveries.

## 2. Materials and Methods

### 2.1. Study Design and Population

This retrospective cohort study was conducted at a single university-affiliated medical centre with approximately 4400 deliveries annually. Data were obtained from the institutional electronic medical record for all eligible births between January 2018 and July 2024. The study was approved by the Institutional Review Board (approval number HYMC-007-26). Given the study’s retrospective nature, the requirement for patient consent was waived.

The cohort included women with live singleton pregnancies in cephalic presentation who delivered vaginally. Multiple gestations and all cesarean deliveries (elective or intrapartum) were excluded.

### 2.2. Exposure Definition

The primary exposure was intrapartum febrile morbidity, defined as the presence of intrapartum fever or a clinical diagnosis of chorioamnionitis. Intrapartum fever was defined as a maternal oral temperature ≥38.0 °C (≥100.4 °F) [12]. Chorioamnionitis was defined if maternal fever was accompanied by leukocytosis ≥15 × 10^9^ cells/L, fetal tachycardia (>160 bpm) and/or purulent fluid from the cervical os [17].

In accordance with institutional practice, in cases of documented fever, a standard laboratory and microbiological workup was performed, including blood cultures and urine cultures. Intravenous antibiotic therapy was subsequently administered according to the clinical scenario, including penicillin for group B streptococcus prophylaxis or broad-spectrum antibiotics for suspected chorioamnionitis [18].

### 2.3. Outcome Definition

The primary outcome was maternal birth trauma. Obstetric lacerations were classified according to standard ACOG definitions [1]. Perineal trauma was defined as any injury to the birth canal sustained during vaginal delivery, including first- through fourth-degree perineal lacerations, cervical lacerations, and episiotomy. Episiotomy was included despite being an iatrogenic incision because it is typically performed selectively when there is concern for impending severe perineal tearing. An intact perineum was defined as the absence of any degree of perineal laceration, cervical laceration, or episiotomy. Perineal lacerations were further grouped as: Low-grade tears (first- and second-degree lacerations) and OASIS (third- and fourth-degree lacerations).

### 2.4. Data Collection

Maternal demographic and baseline clinical variables were extracted from the electronic medical record and included maternal age at delivery, early-pregnancy body mass index (BMI), parity, and relevant comorbidities, including pregestational diabetes, gestational diabetes, and hypertensive disorders.

Obstetric and intrapartum variables included gestational age at delivery, induction of labor (prostaglandin E1 or E2 and/or transcervical balloon catheter), oxytocin augmentation, epidural analgesia, presence of intrapartum fever, duration of the second stage of labor, and mode of vaginal delivery (spontaneous vs. operative, with operative vaginal delivery performed exclusively by vacuum extraction at our institution). Neonatal variables included birth weight, umbilical arterial pH, 5 min Apgar score, and admission to the neonatal intensive care unit (NICU).

### 2.5. Statistical Analysis

Continuous variables were assessed for normality; as most did not follow a normal distribution, they are presented as medians with interquartile ranges [IQRs]. Categorical variables are presented as frequencies and percentages. Univariate analysis was performed using the chi-square or Fisher’s exact test for categorical data and the Mann–Whitney U test for continuous data.

To determine the independent association between intrapartum febrile morbidity and birth trauma, multivariate logistic regression models were constructed for the total cohort and stratified by parity (nulliparous vs. multiparous). Potential confounders were selected a priori based on clinical relevance and previously reported risk factors for birth trauma. The models were adjusted for clinically relevant variables, including maternal age, BMI, gestational age at delivery, epidural analgesia, duration of the second stage of labor, birthweight, and mode of delivery, as well as variables that were statistically significant in the univariate analysis. Results are reported as adjusted odds ratios (aORs) with 95% confidence intervals (CIs). All tests were two-tailed, with a significance level set at *p* < 0.05. All analyses were performed using IBM SPSS Statistics for Windows, version 26.0 (IBM Corp., Armonk, NY, USA). Patients with missing values for BMI were excluded from multivariable analyses requiring this variable (complete-case analysis).

As the study included all eligible vaginal deliveries during the study period, no priori sample size calculation was performed.

## 3. Results

**Figure 1:**
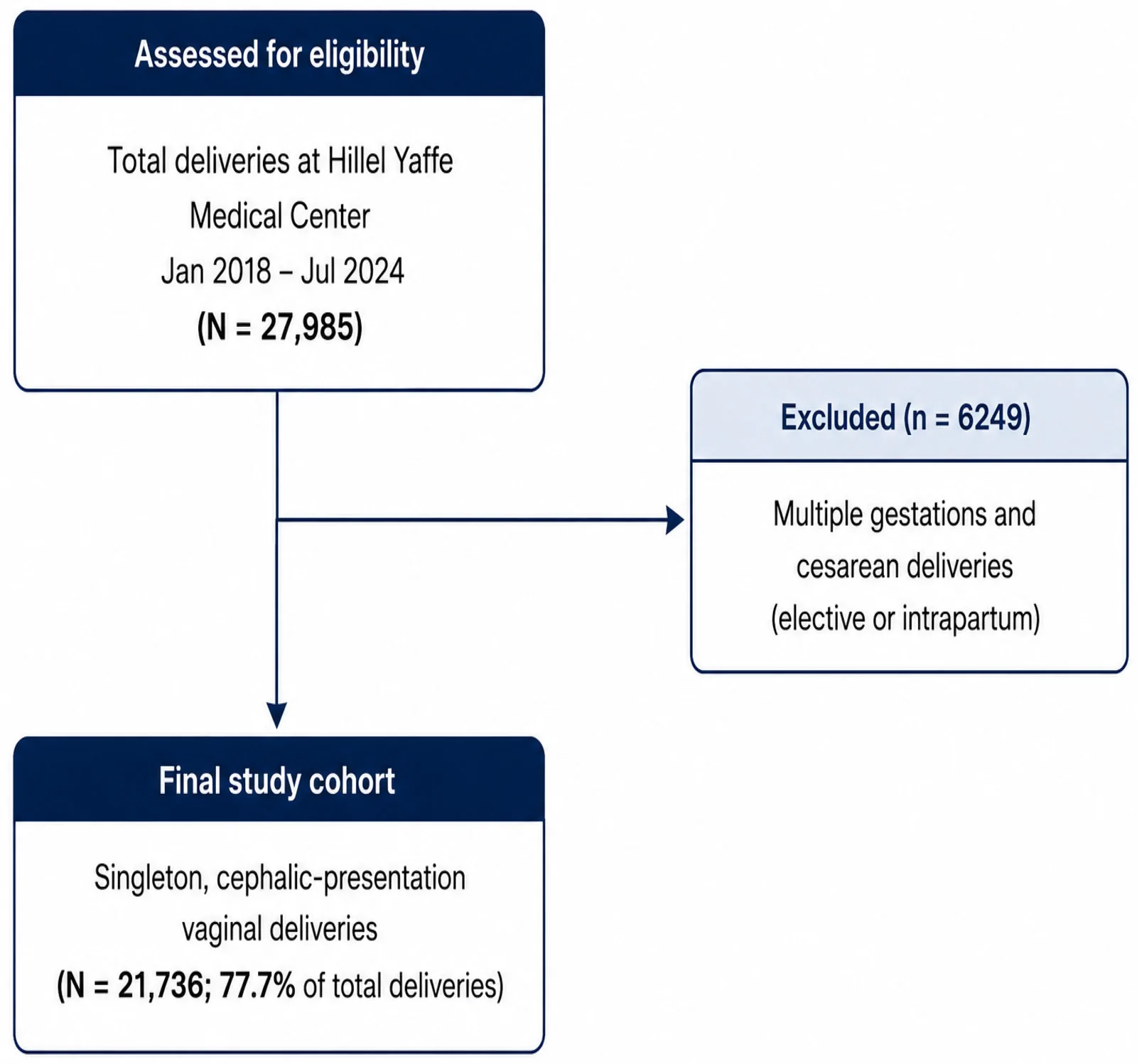
Flow diagram of patient inclusion and exclusion. Of 27,985 deliveries at Hillel Yaffe Medical Center between January 2018 and July 2024, 6249 women were excluded (multiple gestations and cesarean deliveries, elective or intrapartum), yielding a final study cohort of 21,736 women with singleton, cephalic-presentation vaginal deliveries (77.7% of total deliveries).

During the study period, a total of 27,985 deliveries occurred at Hillel Yaffe Medical Center (Figure 1). Following the application of inclusion and exclusion criteria, the final study cohort comprised 21,736 women (77.7% of total deliveries). The baseline demographic and obstetric characteristics of the overall cohort included a median maternal age of 29 years (IQR, 26.0–33.0), a median early-pregnancy BMI of 28.2 kg/m^2^ (IQR, 25.0–32.0), and a median gestational age at delivery of 39.6 weeks (IQR, 38.7–40.3). Nulliparous women accounted for 32.0% (*n* = 6948) of the cohort, and the median neonatal birth weight was 3270 g (IQR, 2992–3558). Within this final study group, 9075 women (41.8%) sustained birth trauma, while 12,661 (58.2%) delivered with an intact perineum. Among women with trauma, 77.1% had first- or second-degree tears, 1.1% experienced OASIS, 27.2% underwent episiotomy, and 1.0% had cervical tears; these categories were not mutually exclusive, as a woman could sustain both a laceration and an episiotomy in the same delivery (Table 1). Women with birth trauma were younger and more likely to be nulliparous (48% vs. 20%, *p* < 0.001). They also had a slightly higher BMI and higher rates of obesity (BMI ≥ 30 kg/m^2^) and hypertensive disorders, whereas the overall prevalence of diabetes did not differ significantly between groups (Table 1).

Compared with women delivering with an intact perineum, those with maternal birth trauma had higher rates of intrapartum febrile morbidity (4.2% vs. 1.8%, *p* < 0.001) and operative delivery (16.6% vs. 5.6%, *p* < 0.001), as well as greater use of oxytocin augmentation and epidural analgesia (both *p* < 0.001). Second-stage duration was significantly longer in the birth trauma group (median 55 min [IQR, 17–126] vs. 19 min [IQR, 9–54], *p* < 0.001). Furthermore, adverse intrapartum and postpartum events, including postpartum hemorrhage (PPH), manual revision of the uterine cavity, and shoulder dystocia, were more frequent among women who sustained maternal birth trauma (all *p* < 0.001). Neonates in the birth trauma group had slightly higher birth weights and lower umbilical arterial pH values, whereas NICU admission rates were similar between the groups (Table 1).

In parity-stratified analyses, among nulliparous women (*n* = 6948), birth trauma was associated with higher rates of operative delivery (26.8% vs. 17.0%, *p* < 0.001), epidural use, oxytocin augmentation (both *p* < 0.001), intrapartum febrile morbidity (6.9% vs. 5.3%, *p* = 0.01), and a longer second stage (112 vs. 93 min, *p* < 0.001). Among multiparous women (*n* = 14,788), birth trauma remained associated with operative delivery (7.0% vs. 2.8%, *p* < 0.001), intrapartum febrile morbidity (1.7% vs. 0.9%, *p* < 0.001), and a longer second stage (19 vs. 14 min, *p* < 0.001) (Table 2).

### Multivariate Logistic Regression Analysis

Multivariate analysis (Table 3) identified nulliparity as the strongest independent predictor of birth trauma in the overall cohort (adjusted odds ratio [aOR] 2.7; 95% confidence interval [CI], 2.4–3.1). Other significant independent risk factors included instrumental delivery (aOR 1.6; 95% CI, 1.4–1.9), intrapartum febrile morbidity (aOR 1.5; 95% CI, 1.1–1.8), and epidural analgesia (aOR 1.3; 95% CI, 1.1–1.4). In parity-specific models, for nulliparous women, the primary drivers of birth trauma were epidural use (aOR 1.8; 95% CI, 1.4–2.1) and operative delivery (aOR 1.5; 95% CI, 1.3–1.9). Intrapartum febrile morbidity was not independently associated with birth trauma in the nulliparous model (aOR 1.3; 95% CI, 0.9–1.7). Among multiparous women, intrapartum febrile morbidity remained an independent predictor of birth trauma (aOR 1.8; 95% CI, 1.2–2.9), alongside operative delivery (aOR 1.9; 95% CI, 1.4–2.5).

## 4. Discussion

Maternal birth trauma represents one of the most common complications of vaginal delivery and remains a major source of short- and long-term postpartum morbidity worldwide. In this large cohort intrapartum febrile morbidity was independently associated with maternal birth trauma after adjustment for established maternal and obstetric risk factors. Although nulliparity and operative vaginal delivery demonstrated the strongest associations, intrapartum febrile remained an independent factor for birth trauma in the overall cohort and among multiparous women. These findings suggest that intrapartum febrile morbidity may identify a labor environment associated with increased risk of lower genital tract injury beyond traditional mechanical risk factors. Given the observational design of this study, these findings should be interpreted as identifying a clinically recognizable marker of increased trauma risk rather than establishing a causal pathway.

Maternal birth trauma, particularly OASIS, has consistently been associated with nulliparity, operative vaginal delivery, fetal birth weight, and prolonged second stage of labor. These variables underpin most contemporary risk stratification models, which primarily incorporate maternal, fetal, and labor-related mechanical variables. In contrast, intrapartum fever or infection has not been routinely incorporated into predictive models, and its independent contribution to perineal trauma remains incompletely defined [14,15,16].

Massalha et al. [19], in a study limited to spontaneous vaginal deliveries, reported that the overall prevalence of perineal tears was comparable between women with intrapartum fever and those who remained afebrile during labor. After adjustment for baseline characteristics, intrapartum fever was not associated with an increased risk of perineal injury, and the adjusted rate of OASIS was lower in the febrile cohort. These findings suggest that fever alone may not independently increase the risk of obstetric trauma.

Conversely, in a retrospective cohort restricted to operative vaginal deliveries, Wilkie et al. [20] reported that chorioamnionitis was independently associated with higher-order perineal lacerations (aOR 2.2; 95% CI, 1.09–4.44). However, the cohort was restricted to operative vaginal deliveries, and the diagnostic criteria for chorioamnionitis were not explicitly specified. It therefore remains unclear whether the reported cases reflected clinically diagnosed chorioamnionitis or a broader intrapartum febrile spectrum as defined in the present study.

Taken together, the existing evidence remains limited and heterogeneous, with discrepancies that likely reflect differences in study populations, including restriction to spontaneous vaginal deliveries or exclusively instrumental births, as well as variation in adjustment for confounding factors. Our findings bridge this gap in the literature by demonstrating an independent association between a comprehensive definition of intrapartum febrile morbidity and maternal birth trauma in a large, unselected cohort that includes both spontaneous and operative vaginal deliveries.

A central finding of this study is the parity-specific pattern in trauma risk. While intrapartum febrile morbidity was a strong independent predictor of trauma in multiparous women (aOR 1.8), it did not retain statistical significance in the nulliparous model. This divergence is hypothesized to reflect differing contributions of mechanical and biochemical pathways of tissue injury; this proposed mechanism remains speculative and requires confirmation in studies incorporating biological markers of inflammation.

In nulliparous women, the overwhelming mechanical demands of a first vaginal birth, characterized by a rigid, previously undistended perineum, likely supersede secondary biochemical factors. The profound physical forces required to distend the nulliparous tissue effectively mask the more subtle effects of inflammation in statistical models.

Conversely, multiparous women possess a previously remodeled and more compliant pelvic floor, establishing a lower baseline risk for spontaneous mechanical tearing. When trauma occurs in multiparous women, it may therefore be more likely to reflect additional modifying factors beyond mechanical stretch alone. Intrapartum febrile morbidity may represent one such clinically recognizable marker. Acute inflammatory processes are involved in extracellular matrix remodeling and tissue repair pathways and may plausibly influence tissue integrity during labor [13]. However, because this study did not include histopathological, microbiological, or molecular assessment of perineal tissue, the observed association should be interpreted as hypothesis-generating rather than evidence of a proven causal mechanism.

From a clinical standpoint, recognizing this inflammatory component is highly relevant. Nulliparity reflects intrinsic, non-modifiable biological factors. Operative vaginal delivery, while strongly linked to injury, is typically reserved for compelling maternal or fetal indications and represents a necessary intervention rather than a readily modifiable exposure. By contrast, intrapartum febrile morbidity represents a potentially modifiable contributor to tissue vulnerability. While causality cannot be definitively established, efforts to reduce the infectious or inflammatory milieu of labor, through the avoidance of unnecessarily prolonged labor, minimizing repetitive vaginal examinations, and the timely administration of targeted antibiotics, may theoretically attenuate tissue edema and preserve collagen integrity; whether this translates into a measurable reduction in obstetric injury remains to be tested prospectively.

Episiotomy was included within the composite outcome of maternal birth trauma despite representing an iatrogenic intervention. At our institution, episiotomy is performed selectively, rather than routinely, and is typically reserved for clinical situations in which substantial perineal strain is anticipated or when expedited delivery is required, such as during operative vaginal birth or in the setting of non-reassuring fetal status. In many cases, the procedure is undertaken when the perineal tissues demonstrate signs of impending uncontrolled tearing or excessive tension, with the aim of directing tissue disruption and potentially limiting more extensive injury. Consequently, the use of episiotomy often reflects an underlying state of heightened mechanical stress or tissue vulnerability at the time of delivery. For this reason, episiotomy was considered appropriate to include within the broader spectrum of clinically relevant birth trauma in the present analysis.

### Strengths and Limitations

Strengths of this study include the large contemporary cohort, detailed intrapartum data from electronic medical records, parity-stratified analyses, and comprehensive multivariable adjustment for established risk factors. Furthermore, adopting a pragmatic, clinically defined category for intrapartum febrile morbidity enhances the generalizability of our findings to routine real-world obstetric practice.

Limitations include the retrospective design and the inherent potential for misclassification of infection, particularly the difficulty in distinguishing true infectious fever from epidural related hyperthermia. In clinical practice, the syndrome of intraamniotic infection (chorioamnionitis) is classically characterized by maternal fever accompanied by inflammatory signs such as maternal or fetal tachycardia, uterine tenderness, or leukocytosis [17]. However, the clinical entity is increasingly recognized as heterogeneous and may reflect both infectious and non-infectious inflammatory processes [21,22]. Intrapartum fever (defined as maternal temperature ≥38.0 °C) remains the sine qua non of the clinical diagnosis, yet accurately distinguishing isolated fever from full clinical chorioamnionitis in real time can be challenging. For this reason, the broader category of intrapartum febrile morbidity was used, defined as any documented maternal temperature ≥38.0 °C during labor, thereby capturing both isolated intrapartum fever and cases meeting clinical criteria for chorioamnionitis. Although this pragmatic approach reflects bedside decision making, it may have resulted in some degree of exposure misclassification. Additionally, histologic confirmation of chorioamnionitis through placental pathology was not assessed, relying instead on the clinical phenotype that guides intrapartum management. Because episiotomy is an operator-driven intervention rather than a passive injury, and is disproportionately represented among nulliparous trauma cases (45.1% vs. 10.3% among multiparous women), its inclusion in the composite birth trauma outcome may introduce confounding by indication that cannot be fully disentangled in the present design. Birth weight was modeled as a continuous variable rather than as a categorical macrosomia threshold; this approach was chosen to preserve statistical power and avoid arbitrary cut-points, though it does not permit direct estimation of a macrosomia-specific effect. The composite exposure of intrapartum febrile morbidity combines isolated fever with clinical chorioamnionitis, and the composite outcome of birth trauma spans first- and second-degree lacerations, OASIS, cervical lacerations, and episiotomy; these subcategories carry different clinical severity and may have divergent relationships with intrapartum inflammation, and OASIS in particular, though rare in this cohort (101 cases), was not analyzed separately by exposure status. Stratified and subgroup analyses were not corrected for multiple comparisons, and the parity-stratified findings should therefore be regarded as exploratory rather than confirmatory. These distinctions were beyond the scope of the current analysis and are proposed as directions for future work with adequately powered subgroups. As with any observational study, residual confounding cannot be entirely excluded and causality cannot be inferred.

## 5. Conclusions

Intrapartum febrile morbidity was independently associated with maternal birth trauma in a large, contemporary cohort of vaginal births, with a particularly pronounced association among multiparous women. These findings suggest that febrile morbidity may be a clinically relevant marker of increased trauma risk beyond established mechanical factors. Prospective studies incorporating microbiological, histopathological, and wound-healing outcomes are needed to clarify whether inflammation contributes causally to tissue vulnerability during childbirth.

## Figures and Tables

**Table 1:** Characteristics of Patients with Birth trauma vs. Intact Perineum.

Characteristic	Birth Trauma (*n* = 9075)	Intact Perineum (*n* = 12,661)	*p* Value
Maternal Characteristics			
Age, y	28.0 [25.0–32.0]	30.0 [26.3–34.0]	<0.001
Young (<18 y)	60 (0.7)	36 (0.3)	<0.001
Advanced age (≥40 y)	249 (2.7)	511 (4.0)	<0.001
BMI, kg/m^2 #^	28.4 [25.4–32.0]	28.0 [25.1–31.6]	<0.001
Obesity (BMI ≥ 30)	1708 (38.7)	2160 (36.3)	0.008
Nulliparity	4397 (48.0)	2551 (20.0)	<0.001
Maternal Comorbidities			
Diabetes mellitus, all types	806 (8.9)	1067 (8.4)	0.125
Gestational diabetes	768 (8.5)	977 (7.7)	0.024
Hypertension, all types	567 (6.2)	588 (4.6)	<0.001
Labor and Delivery			
Gestational age, wk	39.7 [38.9–40.3]	39.4 [38.6–40.3]	<0.001
Intrapartum febrile morbidity	384 (4.2)	232 (1.8)	<0.001
Induction of labor	1356 (14.9)	1478 (11.7)	<0.001
Oxytocin augmentation	5327 (58.7)	6072 (48.0)	<0.001
Epidural analgesia	6881 (75.8)	8365 (66.1)	<0.001
Operative vaginal delivery	1508 (16.6)	715 (5.6)	<0.001
Second-stage duration, min	55 [17–126]	19 [9–54]	<0.001
Shoulder dystocia	108 (1.2)	107 (0.8)	0.005
Birth Trauma Category			
First- or second-degree laceration	6998 (77.1)	—	—
OASIS (third- or fourth-degree)	101 (1.1)	—	—
Episiotomy	2464 (27.2)	—	—
Cervical laceration	85 (1.0)	—	—
Neonatal and Pregnancy Outcomes			
Birth weight, g	3300 [3025–3580]	3250 [2970–3540]	<0.001
Umbilical arterial pH	7.30 [7.24–7.34]	7.33 [7.27–7.37]	<0.001
Cord pH ≤ 7.0	19 (0.2)	16 (0.1)	0.10
5 min Apgar score < 7	27 (0.3)	185 (1.5)	<0.001
NICU admission	491 (5.4)	698 (5.5)	0.70
Postpartum hemorrhage	591 (6.5)	300 (2.4)	<0.001
Uterine cavity revision	339 (3.8)	350 (2.8)	<0.001

Data are presented as median (interquartile range, IQR; 25th–75th percentile) or *n* (%). Birth Trauma Category subgroups are not mutually exclusive (e.g., a woman may have both a laceration and an episiotomy). Hypertension—including chronic hypertension, gestational hypertension, and all types of preeclampsia. ^#^ BMI data were available for 10,612 patients. OASIS—Obstetric Anal Sphincter Injury; NICU—neonatal intensive care unit.

**Table 2:** Parity-stratified comparison of women with and without maternal birth trauma.

	Multiparous (*n* = 14,788)			Nulliparous (*n* = 6948)		
Characteristic	Intact Perineum (*n* = 10,110)	Birth Trauma (*n* = 4678)	*p* Value	Intact Perineum (*n* = 2551)	Birth Trauma (*n* = 4397)	*p* Value
Maternal Characteristics						
Age, y	31.0 [27.0–35.0]	31.0 [27.0–34.0]	<0.001	27.0 [24.0–30.0]	26.0 [24.0–29.0]	0.01
BMI, kg/m^2 #^	28.0 [25.2–31.6]	28.7 [25.7–32.3]	<0.001	28.0 [24.8–31.8]	28.2 [25.1–31.9]	0.25
Obesity (BMI ≥ 30)	1692 (36.4)	855 (40.8)	<0.001	486 (36.1)	853 (36.7)	0.50
Maternal Comorbidities						
Diabetes mellitus, all types	835 (8.3)	418 (8.9)	0.17	232 (9.1)	388 (8.8)	0.60
Gestational diabetes	759 (7.5)	391 (8.4)	0.07	218 (8.5)	377 (8.6)	0.74
Hypertension, all types	434 (4.3)	222 (4.7)	0.21	154 (6.0)	345 (7.8)	0.01
Labor and Delivery						
Gestational age, wks	39.6 [38.6–40.3]	39.7 [38.9–40.4]	<0.001	39.4 [38.4–40.3]	39.6 [38.7–40.3]	<0.001
Intrapartum febrile morbidity	96 (0.9)	79 (1.7)	<0.001	136 (5.3)	305 (6.9)	0.01
Induction of labor	1015 (10.0)	1015 (10.0)	0.42	463 (18.1)	866 (19.7)	0.11
Oxytocin augmentation	4366 (43.2)	2077 (44.4)	0.16	1706 (66.9)	3250 (73.9)	<0.001
Epidural analgesia	6435 (63.6)	3126 (66.8)	<0.001	1930 (75.7)	3755 (85.4)	<0.001
Operative vaginal delivery	282 (2.8)	328 (7.0)	<0.001	433 (17.0)	1180 (26.8)	<0.001
Second-stage duration, min	14 [7–31]	19 [9–48]	<0.001	93 [39–147]	112 [56–163]	<0.001
Birth Trauma Category						
First- or second-degree laceration	—	4214 (90.1)	—	—	2784 (63.3)	—
OASIS (third- or fourth-degree)	—	34 (0.7)	—	—	67 (1.5)	—
Episiotomy	—	480 (10.3)	—	—	1985 (45.1)	—
Cervical laceration	—	34 (0.7)	—	—	51 (1.2)	—
Neonatal Outcomes						
Birth weight, g	3278 [2996–3565]	3380 [3105–3656]	<0.001	3158 [2856–3435]	3220 [2942–3492]	<0.001
Cord pH ≤ 7.0	9 (0.1)	7 (0.2)	0.32	7 (0.3)	12 (0.3)	0.86
5 min Apgar score < 7	111 (1.1)	13 (0.3)	<0.001	74 (2.9)	14 (0.3)	<0.001
NICU admission	495 (4.9)	189 (4.0)	0.02	203 (8.0)	302 (6.9)	0.08

Hypertension—including chronic hypertension, gestational hypertension, and all types of preeclampsia. ^#^ BMI data were available for 10,612 patients. OASIS—Obstetric Anal Sphincter Injury; NICU—neonatal intensive care unit.

**Table 3:** Multivariable logistic regression analysis for factors associated with maternal birth trauma.

Cohort	Variable	Adjusted OR	95% CI
All Cohort (*N* = 21,736)			
	Nulliparity	2.7	2.4–3.1
	Instrumental delivery	1.6	1.4–1.9
	Intrapartum febrile morbidity	1.5	1.1–1.8
	Epidural analgesia	1.3	1.1–1.4
	Hypertension, all types	1.3	1.0–1.5
	Gestational age	1.0	1.0–1.1
	BMI	1.0	0.9–1.0
	Birth weight, g	1.0	1.0–1.0
	Oxytocin augmentation	0.9	0.8–1.0
Nulliparous (*n* = 6948)			
	Age	0.9	0.9–1.0
	Induction of labor	1.0	0.9–1.2
	Second-stage duration	1.0	1.0–1.0
	Epidural use	1.8	1.4–2.1
	Operative delivery	1.5	1.3–1.9
	Intrapartum febrile morbidity	1.3	0.9–1.7
	Gestational age	1.1	1.0–1.1
	Oxytocin augmentation	0.8	0.7–1.0
	Birth weight, g	1.0	1.0–1.0
Multiparous (*n* = 14,788)			
	Age	1.0	0.9–1.0
	BMI	1.0	0.9–1.0
	Hypertension, all types	1.3	1.0–1.7
	Second-stage duration	1.0	1.0–1.0
	Operative delivery	1.9	1.4–2.5
	Intrapartum febrile morbidity	1.8	1.2–2.9
	Epidural use	1.0	0.8–1.2
	Gestational age	1.0	1.0–1.1
	Birth weight, g	1.0	1.0–1.0

Odds ratios are reported per one-unit increase.

## Data Availability

The data supporting the findings of this study are not publicly available due to privacy and ethical restrictions but may be available from the corresponding author upon reasonable request and subject to institutional approval.
